# Cuproptosis-related gene ACAD8 inhibits the metastatic ability of colorectal cancer by inducing cuproptosis

**DOI:** 10.3389/fimmu.2025.1560322

**Published:** 2025-04-03

**Authors:** HuiE Zhuang, Yizhen Chen, Sifu Huang

**Affiliations:** ^1^ Department of Gastroenterology, The Second Affiliated Hospital of Fujian Medical University, Quanzhou, Fujian, China; ^2^ Fujian Medical University, Fuzhou, Fujian, China; ^3^ Fujian Provincial Hospital, Fuzhou University Affiliated Provincial Hospital, School of Medicine, Fuzhou University, Fuzhou, Fujian, China

**Keywords:** colorectal cancer, cuproptosis, ACAD8, immunotherapy, distant metastasis

## Abstract

**Background:**

Distant metastasis of colorectal cancer (CRC) significantly impacts patient prognosis. Cuproptosis is a new form of copper ion-dependent cell death. However, whether cuproptosis-related genes (CRGs) play a role in the metastatic potential of CRC remains unclear. This study focuses on CRGs-ACAD8 to explore its role and mechanism in metastatic CRC (mCRC).

**Methods:**

Clinical sample data from TCGA, GEO, and Fujian Provincial Hospital patients were integrated to analyze ACAD8 expression and its association with the diagnosis and prognosis of CRC. Small interfering RNA, immunohistochemistry, colony formation, wound-healing assays and so on were used to evaluate the biological functions of ACAD8. Bioinformatics was applied to investigate its relationships with immune infiltration, chemotherapy sensitivity, and signaling pathways.

**Results:**

ACAD8 expression was significantly reduced in mCRC and demonstrated excellent diagnostic performance. Patients with high ACAD8 expression had significantly better survival. ACAD8 was closely associated with immune cell infiltration, and enhanced chemotherapy sensitivity. Pathway enrichment analysis suggested that ACAD8 might inhibit the metastasis of CRC by regulating pathways such as response to metal ions and tight junction organization. Finally, experiments confirmed a positive correlation between copper levels and ACAD8 mRNA expression, with CuCl_2_ upregulating ACAD8 expression. Knockdown of ACAD8 induced cuproptosis. CuCl_2_ inhibited the proliferation, stemness, and migratory abilities of CRC cells, while si ACAD8 attenuated these effects. Moreover, CuCl_2_ enhanced the sensitivity of CRC cells to oxaliplatin and 5-fluorouracil, whereas si ACAD8 diminished this chemosensitizing effect.

**Conclusion:**

As a novel tumor suppressor, low expression of CRGs-ACAD8 is associated with the metastasis of CRC. ACAD8 holds potential diagnostic and prognostic value and may contribute to the precise treatment of CRC by regulating immune infiltration and chemotherapy sensitivity.

## 1 Background

Colorectal cancer (CRC) is among the most common malignancies globally, with distant metastasis, particularly to the liver and lungs, being a primary factor affecting patient outcomes (1). The metastatic potential of CRC is closely linked to several molecular signaling pathways (2). Additionally, the tumor microenvironment (TME) plays the vital role in promoting the metastasis of cancer (3). Despite considerable research advances, precise treatments for metastatic CRC (mCRC) remain challenging.

Uncovering mechanisms underlying the metastasis of CRC is key to improving treatment strategies. For mCRC patients, combined therapies such as chemotherapy, and immunotherapy have demonstrated efficacy. However, these treatments are often hindered by tumor heterogeneity and resistance (4, 5). Thus, future research should focus on elucidating metastatic mechanisms and developing novel biomarkers to enhance the survival rates of mCRC.

Cuproptosis, a new form of copper-dependent cell death, is intricately linked to intracellular copper ion concentrations (6). Excess copper ions can accumulate and induce cell death by promoting aggregation of lipoylated protein in the mitochondrial tricarboxylic acid cycle (7). Studies have shown significantly elevated copper levels in gastric cancer, where copper induces the lactylation of METTL16 at K229, leading to cuproptosis (8). Additionally, targeting ferroptosis and cuproptosis simultaneously using ferroptosis inducers and copper ionophores has shown promise in the treatment of hepatocellular carcinoma (9). However, few studies have explored cuproptosis mechanisms in CRC (10). The potential role of cuproptosis and cuproptosis-related genes (CRGs) in mCRC has garnered increasing interest as a critical anticancer strategy.

Given the novel insights that cuproptosis provides for cancer research, we hypothesize that CRGs may inhibit the metastasis of CRC. To investigate, we integrated bioinformatics and basic biological experiments to examine the clinical application and underlying mechanisms of CRGs-ACAD8 in CRC. We screened mCRC and cuproptosis-related datasets from the GEO and identified the novel CRG-ACAD8. In the context of cancer research, ACAD8 has been associated with a favorable prognosis in lung adenocarcinoma (LUAD), where it is considered a tumor-associated fibroblast-related gene associated with good survival outcomes (11). Furthermore, ACAD8 has been identified as a novel gene associated with prognosis and immunotherapy through amino acid metabolism pathways in LUAD (12). However, its functional role in CRC remains poorly characterized.

Using TCGA, we analyzed ACAD8 expression differences in mCRC and validated the findings with GEO and tumor samples from CRC patients at Fujian Provincial Hospital. ACAD8 protein levels were analyzed through the Human Protein Atlas (HPA) and verified via immunohistochemistry (IHC). The diagnostic or prognostic value of ACAD8 was evaluated by TCGA, GEO, and patient data (13). We further explored the correlation between ACAD8 expression, immune cell infiltration, immune checkpoint genes, and chemotherapy sensitivity. Pathway enrichment analysis highlighted the potential significance of ACAD8 in the metastasis of CRC. Finally, cell phenotype experiments demonstrated the ability of CRGs-ACAD8 to suppress the metastasis of CRC.

## 2 Materials and methods

### 2.1 CRC samples and data collection

Data sets of mCRC were obtained from the GEO for analysis. Additionally, data from TCGA were cross-validated with GEO findings. CRC patient data from Fujian Provincial Hospital were utilized to validate the results of these two databases.

### 2.2 Cell culture

The human CRC cell line RKO was obtained from Haixing Bioscience Co., Ltd. (China). RKO cells were cultured in medium containing 10% fetal bovine serum (FBS) and 1% antibiotics in a humidified incubator at 37°C with 5% CO_2_.

### 2.3 Cell Counting Kit-8 assay

RKO cells were digested with trypsin and centrifuged. The cells were resuspended in complete medium and seeded into 96-well plates. Each experimental group included five replicate wells. A total of 10μL of CCK-8 solution was added to each well, and optical density (OD) at 450 nm was measured using the microplate reader (13).

### 2.4 Colony-formation assay

Logarithmic-phase RKO cells were digested with trypsin, centrifuged, and resuspended. RKO cells were seeded at a density of 400 or 450 cells per well in six-well plates. After visible colonies formed, cells were fixed and stained with crystal violet. Colonies were photographed and counted. Each experimental group included three replicate wells.

### 2.5 Scratch wound assay

RKO cells were seeded into six-well plates at 99% confluence. A sterile pipette tip was used to create a straight scratch (“wound”). Debris was removed with sterile 1x PBS. Initial scratch images were captured using an inverted microscope. After 48 hours of incubation, additional images were taken, and Image J was used to quantify wound closure to evaluate the migration ability of cell.

### 2.6 RT-qPCR

The amplification of PCR and cycle threshold (CT) determination were performed using the Roche LightCycler 480 system. Reagents for reverse transcription and amplification were provided by Fujian HeRui Biotechnology Co., Ltd (14). The primer sequences for ACAD8 were: Forward sequence: 5’-ATGAGGAGGAGGAGGAGAGG-3’; Reverse sequence: 5’-TTTAGTTTGTCCACCTCTGC-3’.

### 2.7 Small interfering RNA

RKO cells were seeded into six-well plates. When cells reached 65% confluence, they were transfected with siRNAs. Briefly, 200μL of diluted Lipofectamine 3000 was mixed with 200μL of diluted plasmid DNA. After mixing, the mixture was incubated at room temperature for 30 minutes to ensure complete encapsulation. A total of 400μL of the lipid-DNA complex was added to the cells and incubated for 12 hours. The forward sequence of si ACAD8 was: 5’-GGAGAGGAAGGAGAAGTTA-3’; Reverse sequence: 5’-UCAUUCUCCUUCUUCUCCAA-3’.

### 2.8 Immunohistochemistry

CRC tissue samples were fixed, dehydrated, paraffin-embedded, and sectioned. Sections were incubated at 60°C for 12 hours, followed by deparaffinization, hydration, and antigen retrieval. Anti-ACAD8 antibody (Product ID: CSB-PA892457LA01HU, CUSABIO, China) was added and incubated at 4°C for 12 hours. Secondary antibodies were added for incubation, and diaminobenzidine (DAB) was used for staining.

### 2.9 Western blot

Protein samples were extracted from cells, subjected to electrophoresis, transferred to membranes, and blocked. Anti-ACAD8 or Anti-DLAT antibody was incubated at 4°C for 8 hours, followed by secondary antibodies (goat anti-rabbit IgG or goat anti-mouse IgG) for 2 hours. Bands were visualized using ECL substrate and an imaging system. Anti-DLAT antibody was purchased from CST (Catalog #12362).

### 2.10 Diagnostic and prognostic value of ACAD8 in CRC

ROC curves and area under the curve (AUC) values were analyzed and visualized using the R software packages pROC (version 1.18.0) and ggplot2 (version 3.3.6) (14). Survival data from the TCGA database were analyzed using the survival (version 3.3.1) and survminer (version 0.4.9) packages (13).

### 2.11 Analysis of immune cell infiltration and immune checkpoint genes

The ssGSEA algorithm from the GSVA package (version 1.46.0) was used. Spearman correlation analysis was performed to investigate the relationship between ACAD8 expression, immune cell infiltration, and immune checkpoint-related genes.

### 2.12 Chemotherapy sensitivity

Using the GDSC, the impact of ACAD8 expression on chemotherapy response of CRC was analyzed (15). Ridge regression was applied to determine IC50 values using the pRRophetic package.

### 2.13 Pathway enrichment analysis

Differentially expressed genes (DEGs) associated with ACAD8 were analyzed. Functional annotation and pathway enrichment were performed using the clusterProfiler package (version 4.4.4) and visualized using ggplot2 (version 3.3.6).

### 2.14 Copper Assay

A Copper Assay Kit (Abcam, ab272528, UK) was used to extract tumor tissues from 80 CRC patients. The assay followed the protocol of manufacturer, with OD measured at 359 nm.

### 2.15 Statistical analysis

**Figure 1:**
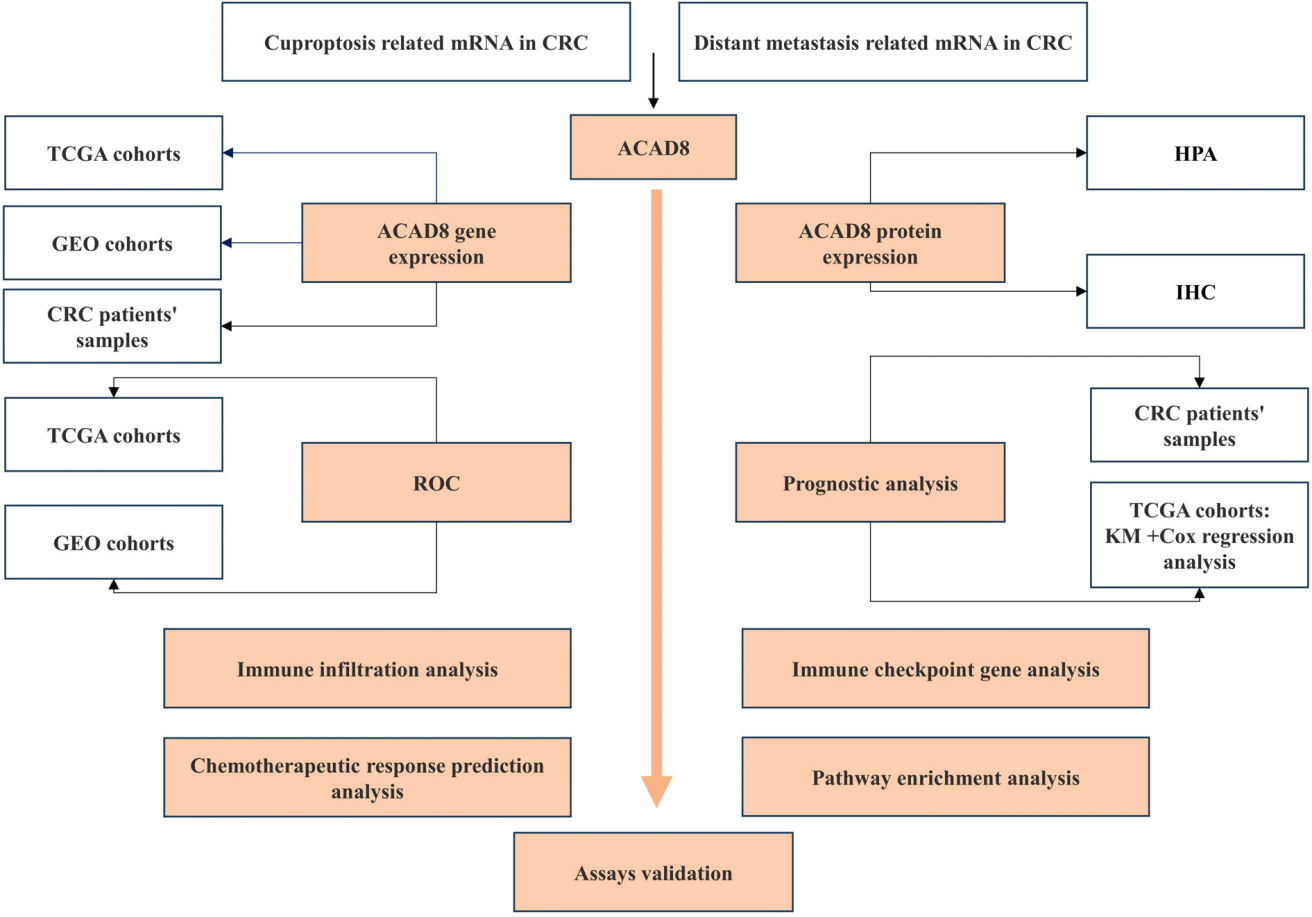
Study flowchart. This illustrates the design and related assays of this study.

Data analysis was performed using R software, GraphPad Prism (version 9.5.1), and SPSS (version 26). Depending on data distribution, statistical analysis included the Wilcoxon rank-sum test, Student’s t test, or chi-square test. Kaplan-Meier (KM) analysis was used to evaluate survival. The study workflow was shown in **Figure 1**.

## 3 Results

### 3.1 Identification of ACAD8 as a crucial CRGs in mCRC

**Figure 2:**
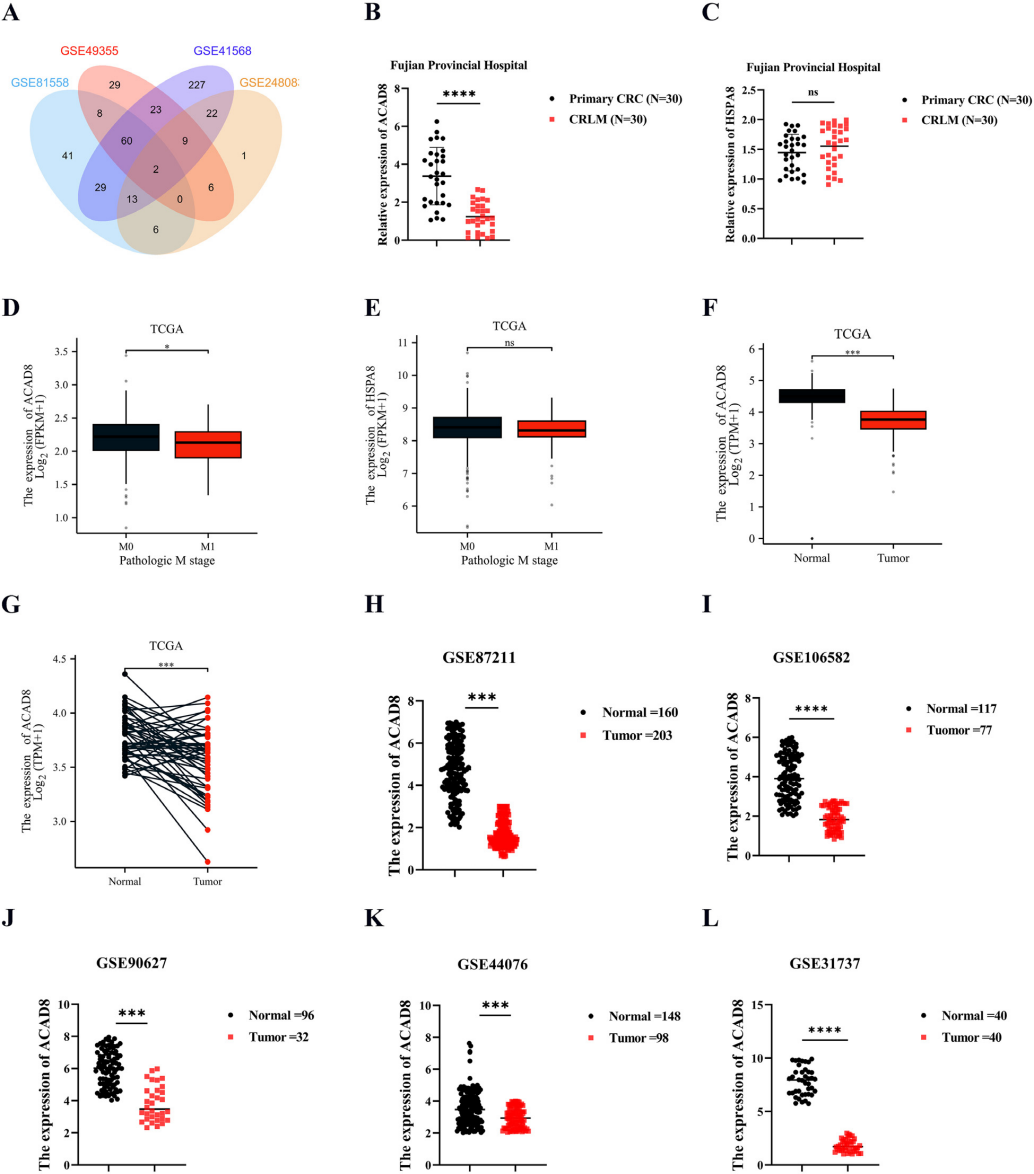
The RNA levels of ACAD8 in CRC. **(A)** ACAD8 was identified by intersecting Venn diagrams after searching the GEO dataset. **(B)** Analyzing the RNA levels of ACAD8 in primary CRC and matched CRLM based on data from Fujian Provincial Hospital patients. **(C)** Analyzing the RNA levels of HSPA8 in primary CRC and matched CRLM based on data from Fujian Provincial Hospital patients. **(D)** The expression difference of ACAD8 among CRC patients in different M stage. **(E)** The expression difference of HSPA8 among CRC patients in different M stage. **(F)** Analyzing the RNA levels of ACAD8 in CRC tissues and normal intestinal epithelial tissues based on TCGA. **(G)** Analyzing the RNA levels of ACAD8 in CRC tissues and matched normal intestinal epithelial tissues based on TCGA. Analyzing the RNA levels of ACAD8 in CRC tissues and normal intestinal epithelial tissues based on GSE87211 **(H)**, GSE106582 **(I)**, GSE90627 **(J)**, GSE44076 **(K)**, and GSE31737 **(L)**. (P>0.05, ns. nonsignificant; P < 0.05 *; P < 0.001 ***; P < 0.0001 ****; analyses were performed using Student’ s t test or Wilcoxon rank-sum test, respectively).

Through an analysis of classic mCRC datasets from GEO (GSE81558, GSE49355, and GSE41568) and cuproptosis-related mRNA (GSE248083), we identified two CRGs associated with the metastasis of CRC: ACAD8 and HSPA8 (**Figure 2A**). To investigate which CRGs plays a central role in metastasis, RNA was extracted from paired primary CRC and liver metastasis samples at Fujian Provincial Hospital. qPCR revealed significantly reduced ACAD8 expression in liver metastasis compared to primary CRC (**Figure 2B**), while HSPA8 showed no significant difference (**Figure 2C**). TCGA data further confirmed that ACAD8 expression is markedly decreased in mCRC (**Figures 2D, E**). Thus, ACAD8 was selected for further study.

To evaluate ACAD8 expression in CRC, we analyzed TCGA data comparing normal intestinal epithelium and CRC tissues, revealing significantly higher ACAD8 expression in normal tissues (**Figure 2F**). Paired CRC and normal epithelium samples confirmed this finding (**Figure 2G**). GEO datasets supported this result, demonstrating consistently higher ACAD8 expression in normal tissues than in CRC (**Figures 2H–L**, **Supplementary Figures 1A–C**). Collectively, these findings suggest that ACAD8 acts as a protective factor, with its mRNA levels significantly reduced in CRC.

After confirming ACAD8 expression in CRC, we examined whether there were also expression differences in pan-cancer. TCGA data indicated that ACAD8 was downregulated in 19 types of cancers and upregulated in 5 (**Supplementary Figure S1D**). Paired sample analysis across pan-cancer also indicated differential expression of ACAD8 in 14 cancer types (**Supplementary Figure S1E**). These results strongly suggest that ACAD8 acts as a tumor suppressor not only in CRC but also in many other cancers.

**Figure 3:**
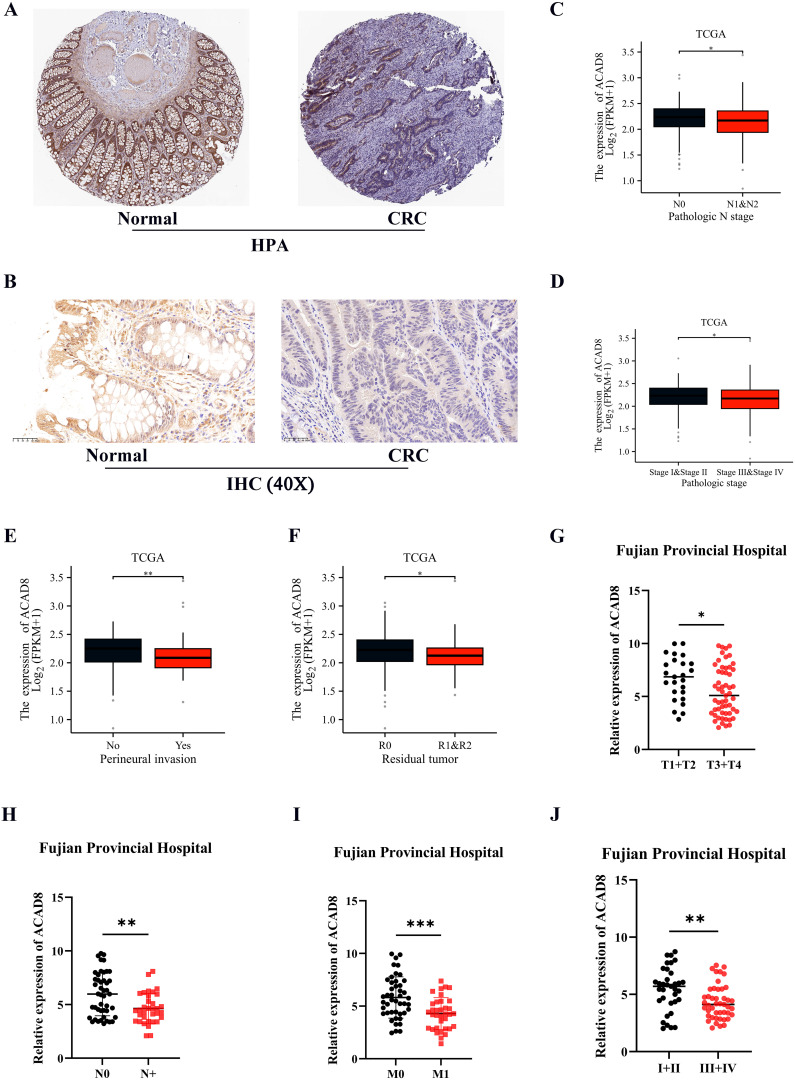
The protein levels of ACAD8 in CRC and association with clinical information. **(A)** Detected the protein expression of ACAD8 in CRC tissues and normal tissues based on the HPA database. **(B)** The protein expression of ACAD8 in CRC tissues and normal tissues using IHC based on patient samples. Based on TCGA, the expression difference of ACAD8 among CRC patients in different N **(C)**, TNM stages **(D)**, perineural invasion **(E)** and residual tumor **(F)**, respectively. Based on data from Fujian Provincial Hospital patients (n=80 CRC patients), the expression difference of ACAD8 among CRC patients in different T **(G)**, N **(H)**, M **(I)**, and TNM stages **(J)**, respectively. (P>0.05, ns. nonsignificant; P < 0.05 *; P < 0.01 **; P < 0.001 ***; analyses were performed using Student’ s t test or Wilcoxon rank-sum test, respectively).

In addition to mRNA levels, we sought to determine if the protein expression of ACAD8 was also reduced in CRC. Results from the HPA database confirmed that ACAD8 protein expression was significantly lower in CRC (**Figure 3A**). This was further validated by IHC of patient samples from Fujian Provincial Hospital (**Figure 3B**). Together, these results demonstrate that both the RNA and protein levels of ACAD8 are significantly higher in normal intestinal epithelial tissues. Furthermore, the correlation between ACAD8 expression and clinical data was discussed. The low expression of ACAD8 was associated with advanced N and TNM stages (**Figures 3C, D**), confirming a correlation between ACAD8 and metastasis of CRC. Additionally, low expression of ACAD8 was also linked to positive surgical margins and nerve invasion (**Figures 3E, F**). Clinical data from Fujian Provincial Hospital confirmed the correlation between ACAD8 expression and clinical stages as observed in TCGA (**Figures 3G–J**).

### 3.2 ACAD8 as a novel diagnostic gene

**Figure 4:**
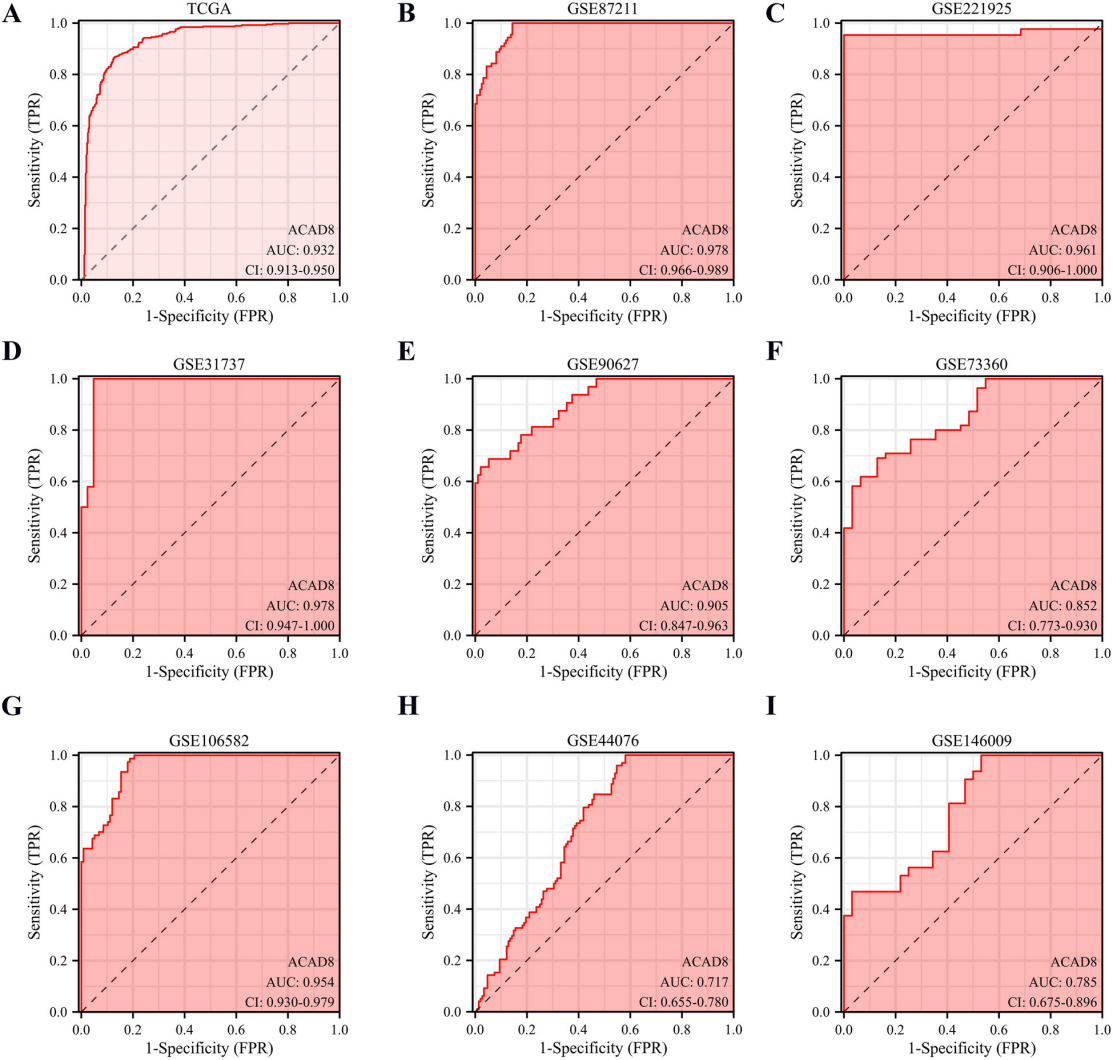
The diagnostic potential of ACAD8 for CRC. **(A)** Based on TCGA, the diagnostic capability of ACAD8 for distinguishing CRC patients from normal individuals. Based on GSE87211 **(B)**, GSE221925 **(C)**, GSE31737 **(D)**, GSE90627 **(E)**, GSE73360 **(F)**, GSE106582 **(G)**, GSE44076 **(H)** and GSE146009 **(I)**, the diagnostic capability of ACAD8 for distinguishing CRC patients from normal individuals.

We investigated whether ACAD8 could serve as a diagnostic biomarker for CRC. ACAD8 could efficiently diagnose CRC (**Figure 4A**, AUC = 0.932). To cross-validate the results from a single database, we analyzed GEO datasets on normal intestinal epithelial tissues and CRC. The results supported that ACAD8 could effectively distinguish between normal tissues and CRC (**Figures 4B–I**, AUC values were 0.978, 0.961, 0.978, 0.905, 0.852, 0.954, 0.717, and 0.785).

### 3.3 ACAD8 as a novel prognostic gene

**Figure 5:**
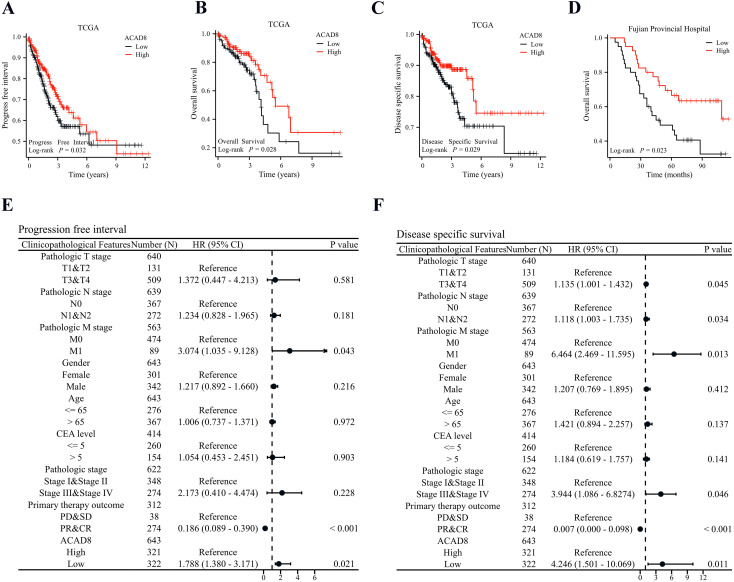
Low expression of ACAD8 indicates poor prognosis in CRC patients. **(A)** Based on TCGA, KM survival curve analysis of the impact of ACAD8 expression on PFI in CRC patients. **(B)** Based on TCGA, KM survival curve analysis of the impact of ACAD8 expression on OS in CRC patients. **(C)** Based on TCGA, KM survival curve analysis of the impact of ACAD8 expression on DSS in CRC patients. **(D)** Based on the data from Fujian Provincial Hospital patients, KM survival curve analysis of the impact of ACAD8 expression on OS in CRC patients. **(E)** A forest plot showing cox regression analysis of the impact of ACAD8 on PFI in CRC. **(F)** A forest plot showing cox regression analysis of the impact of ACAD8 on DSS in CRC.

We explored whether ACAD8 could predict the prognosis of CRC. Using survival data from TCGA patients, we plotted KM curves, which showed that ACAD8 was associated with a better progression-free interval (PFI) and overall survival (OS) (**Figures 5A, B**). These results were further validated after excluding non-tumor-related deaths (**Figure 5C**). To cross-validate the findings, we analyzed the prognostic data of CRC patients from Fujian Provincial Hospital. The results confirmed that ACAD8 expression was associated with improved OS (**Figure 5D**). Cox regression analysis adjusted for confounding factors revealed that ACAD8 expression was significantly correlated with better PFI, disease-specific survival (DSS), and OS (**Figures 5E, F**, **Supplementary Figure S2**).

### 3.4 ACAD8 enhances immune infiltration in CRC

**Figure 6:**
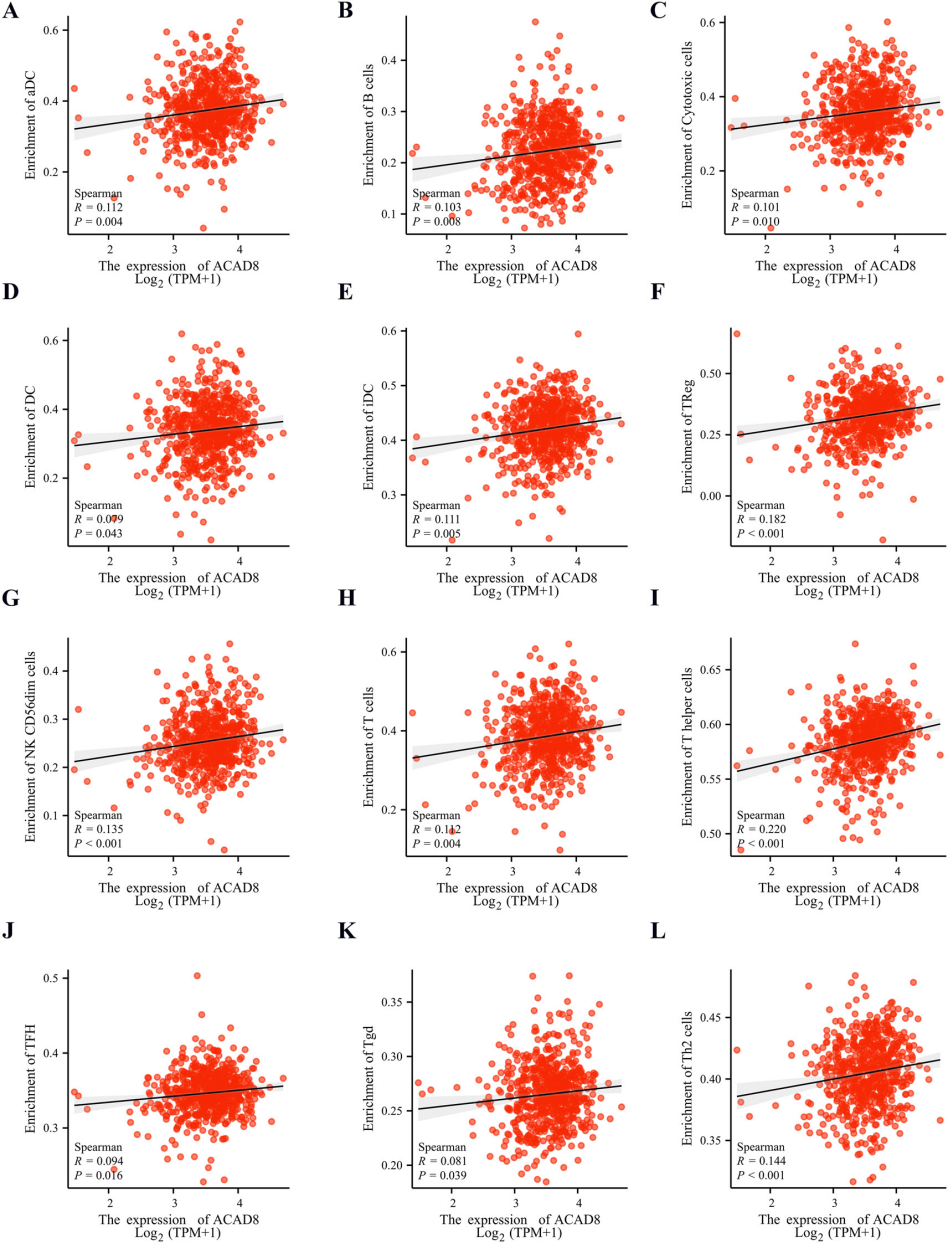
The relationship between expression levels of ACAD8 and immune cell infiltration. The correlation of expression levels of ACAD8 with aDC **(A)**, B cell **(B)**, cytotoxic cell **(C)**, DC **(D)**, iDC **(E)**, Treg **(F)**, cd56dim cell **(G)**, T cell **(H)**, T helper **(I)**, TFH **(J)**, Tgd **(K)**, TH2 cells **(L)**.

The relationship between immune infiltration and tumor metastasis is a complex dynamic balance, and precise regulation of the TME is an important strategy for inhibiting tumor metastasis (16, 17). Spearman analysis revealed that ACAD8 was not correlated with 7 types of immune cells, including mast cells and NK cells (**Supplementary Figures S3A–H**). However, pDC cells showed a negative correlation with ACAD8 (**Supplementary Figure S3I**). On the other hand, B cells and DC cells showed a positive correlation with ACAD8 expression (**Figures 6A–L**). We further analyzed the correlation between ACAD8 expression and immune cell infiltration across pan-cancer datasets (**Supplementary Figure S4**).

### 3.5 ACAD8 improves the response of CRC to chemotherapy

Immune checkpoint inhibitors (ICIs) have become an indispensable part of mCRC (18). Therefore, we explored whether ACAD8 is associated with immune checkpoint-related genes. After extracting 9 representative immune checkpoint markers from TCGA, we found that, except for SIGLEC15, all other markers showed a positive correlation with ACAD8 expression (**Supplementary Figures S5A–I**).

**Figure 7:**
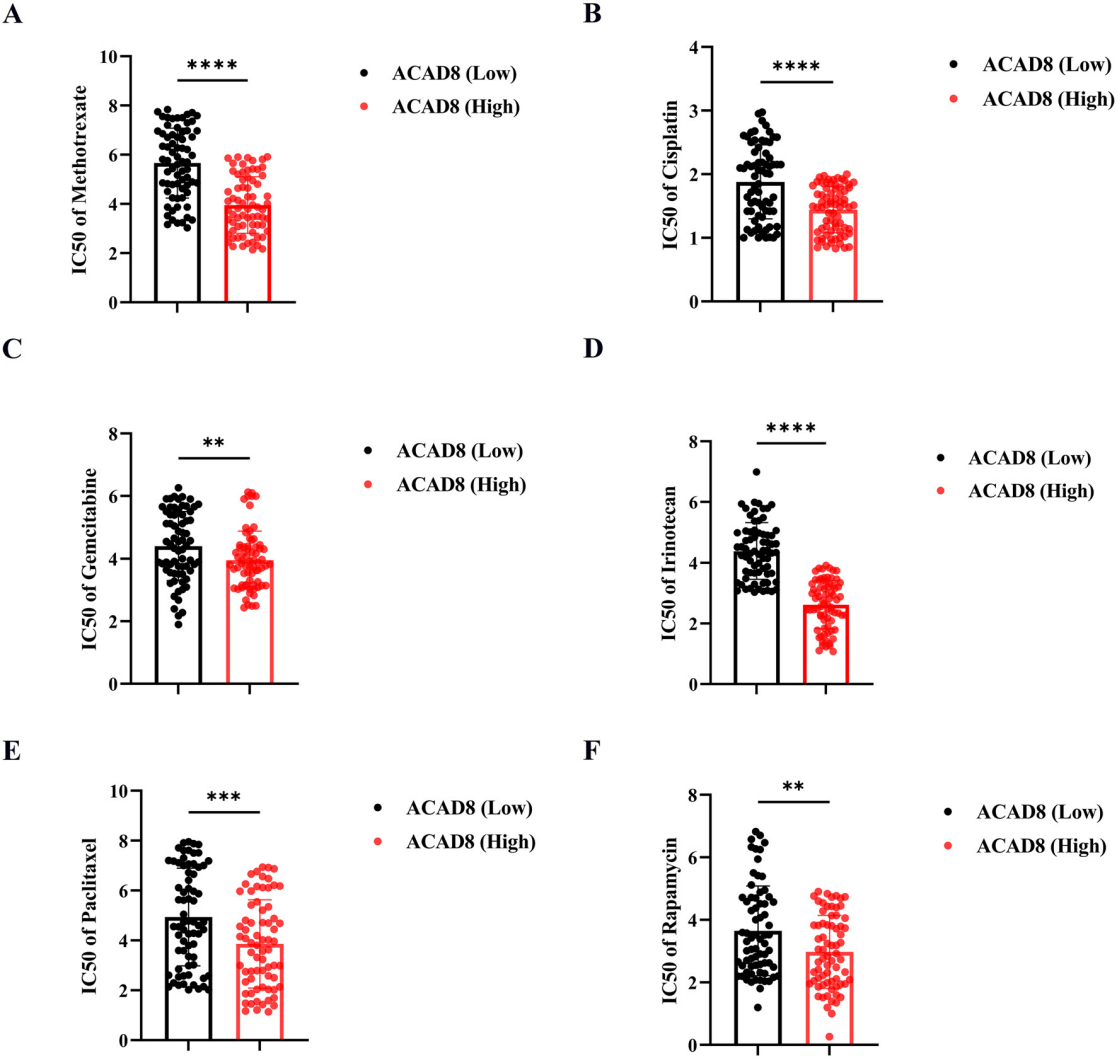
The correlation between ACAD8 and chemotherapy sensitivity in CRC. IC50 of response to Methotrexate **(A)**, Cisplatin **(B)**, Gemcitabine **(C)**, Irinotecan **(D)**, Paclitaxel **(E)**, and Rapamycin **(F)** between the high and low expression of ACAD8 groups. (P>0.05, ns. nonsignificant; P < 0.01 **; P < 0.001 ***; P < 0.0001 ****; analyses were performed using Student’ s t test or Wilcoxon rank-sum test, respectively).

Previous studies have confirmed that copper ions can inhibit tumor chemotherapy resistance in other cancer types (19). In addition to predicting the efficacy of ICIs, we explored whether ACAD8 could enhance the sensitivity to chemotherapy drugs in CRC patients. By analyzing the GDSC database on classic chemotherapy drugs for CRC, we found that high ACAD8 levels were associated with lower IC50 values for 5-Fluorouracil, Cisplatin, Irinotecan, Gemcitabine, Rapamycin, and Paclitaxel (**Figures 7A–F**), suggesting that ACAD8 can increase sensitivity to chemotherapy in CRC.

### 3.6 Functional enrichment analysis of ACAD8

**Figure 8:**
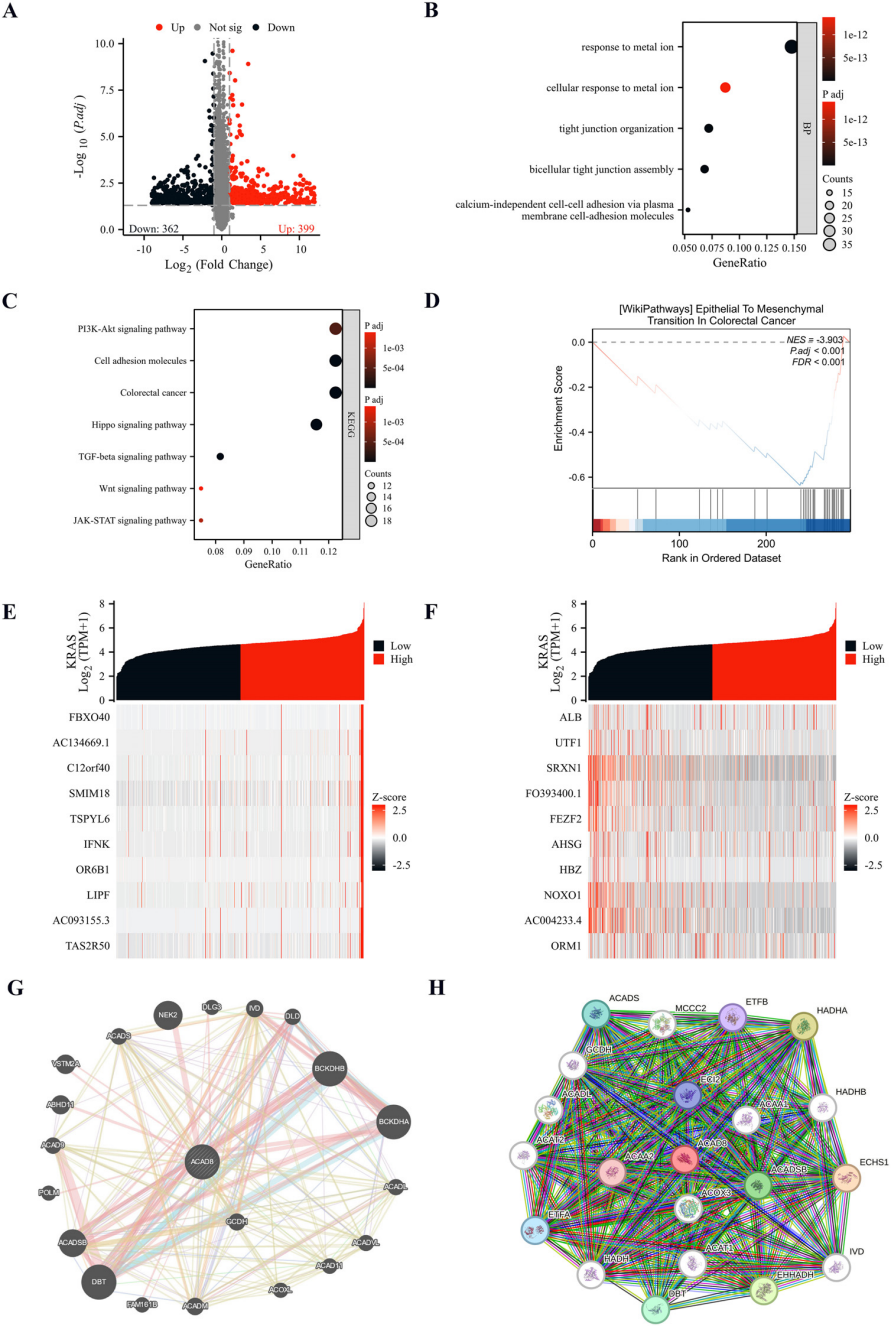
Pathway enrichment analysis of ACAD8 in CRC. **(A)** Volcano plot of ACAD8-related DEGs. **(B)** GO analysis of BP by ACAD8-related DEGs. **(C)** KEGG analysis of pathways by ACAD8-related DEGs. **(D)** GSEA analysis of functions affected by expression differences of ACAD8. **(E, F)** Analysis of co expressed genes related to ACAD8. **(G)** Gene interaction network diagram showing genes interacting with ACAD8 analyzed by Gene Mania. **(H)** Protein-protein interaction (PPI) network analysis of proteins interacting with ACAD8.

After confirming the role of ACAD8 in diagnosis, prognosis, immune infiltration, and drug response of CRC, we hypothesized potential mechanisms by which ACAD8 exerts its effects. Using TCGA data, we simulated the effects of overexpression or knockdown of ACAD8. Differential gene expression analysis revealed 761 significantly associated differentially expressed genes (DEGs) (|FC| > 1, p.adj < 0.05), with 399 genes upregulated and 365 genes downregulated (**Figure 8A**). Further annotation and enrichment analysis of these DEGs indicated that ACAD8 is involved in response to metal ion, cellular response to metal ion, tight junction organization and bicellular tight junction assembly (**Figure 8B**). KEGG analysis revealed that ACAD8 is associated with several signaling pathways, including the PI3K-Akt signaling pathway, Cell adhesion molecules, Colorectal cancer and Hippo signaling pathway (**Figure 8C**). These results were confirmed by GSEA analysis (**Figure 8D**). Additionally, TCGA data mining identified genes whose expression correlated with ACAD8. The results suggested that the expression of ACAD8 was positively correlated with FBXO40, AC134669.1, C12orf40, SMIM18, TSPYL6, IFNK, OR6B1, LIPF, AC093155.3 and TAS2R50 (**Figure 8E**). While ACAD8 was negatively correlated with ALB, UTF1, SRXN1, FO393400.1, FEZF2, AHSG, HBZ, NOXO1, AC004233.4, and ORM1 expression. While ACAD8 was negatively correlated with ALB, UTF1, SRXN1, FO393400.1, FEZF2, AHSG, HBZ, NOXO1, AC004233.4, and ORM1 expression (**Figure 8F**). These suggesting that these may be potential targets through which ACAD8 inhibits metastasis of CRC.

We also investigated potential interacting proteins of ACAD8. We applied GeneMANIA for a comprehensive analysis. The interaction network suggested that DBT, BCKDHA and ACADSB closely interacted with ACAD8 (**Figure 8G**). In addition, we further explored the possibility of interacting proteins through the STRING database, and the protein-protein interaction (PPI) network finally found that ACAA2, ACAA1, ACOX3 and other proteins interacted with ACAD8 (**Figure 8H**). Combined analysis of the above cooperating proteins suggests that DBT and ACADSB may be the potential interaction proteins of ACAD8 in inhibiting the metastasis and invasion of CRC.

### 3.7 Verification the impact of ACAD8 on metastasis of CRC

**Figure 9:**
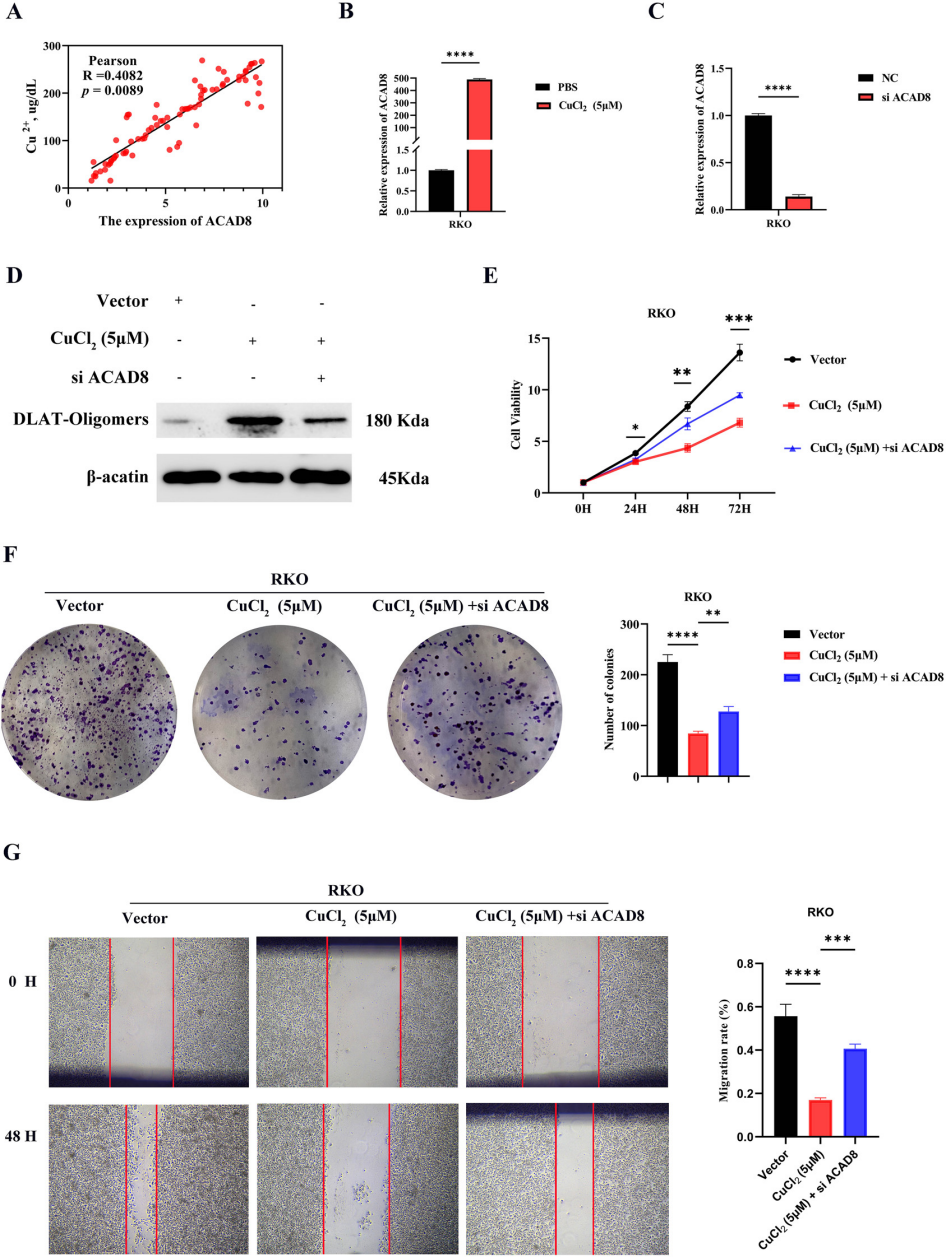
Knockdown of CRGs-ACAD8 promoted the metastatic ability of CRC. **(A)** Analysis of the correlation between Cu^2+^ concentration and ACAD8 expression in CRC. **(B)** RT-qPCR was used to observe the expression of ACAD8 in CRC cells treated with CuCl_2_. **(C)** RT-qPCR confirmed the knockdown of ACAD8. **(D)** WB was used to observe the changes of cuproptosis markers in CRC cells treated with CuCl_2_ and si ACAD8. **(E)** CCK8 assay detected the effect of CuCl_2_ and si ACAD8 on proliferation of CRC. **(F)** Colony formation assay detected the effect of CuCl_2_ and si ACAD8 on stemness capability of CRC. **(G)** The scratch wound assay confirmed the effect of CuCl_2_ and si ACAD8 on migration ability of CRC. (P>0.05, ns. nonsignificant; P < 0.05 *; P < 0.01 **; P < 0.001 ***; P < 0.0001 ****; analyses were performed using Student’s t test, Wilcoxon rank-sum test, One-way analysis of covariance or Kruskal-Wallis test, respectively).

Based on the bioinformatics findings and patient sample validations, we hypothesize that CRGs-ACAD8 may inhibit the metastasis of CRC through cuproptosis. To confirm ACAD8 as a CRG, we correlated copper levels with ACAD8 mRNA expression in CRC, finding a positive correlation (**Figure 9A**). We then investigated whether copper ions could upregulate ACAD8 expression. After treating CRC cells with CuCl_2_, we observed increased ACAD8 expression, suggesting that ACAD8 might be a key regulator in copper ion-induced cuproptosis (**Figure 9B**). Next, we assessed whether ACAD8 could induce cuproptosis. Given that protein lipidation is a central factor in cuproptosis, we used lipidated protein aggregation by dihydrolipoamide S-acetyltransferase (DLAT) as an indicator of cuproptosis (20). The expression level of ACAD8 was inhibited by siRNA, and the success of knocking down ACAD8 was confirmed by RT-qPCR (**Figure 9C**). WB showed that CuCl_2_ activated cuproptosis. However, inhibition of ACAD8 resulted in decreased protein aggregation of the cuproptosis marker DLAT (**Figure 9D**). The above experiments support that as CRGs, ACAD8 can induce cuproptosis.

The above experiments confirmed that as CRGs, ACAD8 can induce cuproptosis. However, whether ACAD8 can inhibit metastasis requires further experimental verification. Cell proliferation provides the cell base for metastasis, cell stemness determines the survival ability of CRC cells in heterogeneous environments, and migration ability is the core driving force for tumor metastasis (21, 22). These three factors work together to constitute the key link of tumor metastasis. Therefore, we subsequently verified the effects of cuproptosis and si ACAD8 on the metastasis ability of CRC through CCK8, colony formation, and scratch wound assay. CCK8 showed that CuCl_2_ inhibited the proliferation of CRC, and si ACAD8 inhibited the effect of CuCl_2_ (**Figure 9E**). The colony formation assay also found that compared with PBS, CuCl_2_ indeed inhibited the stemness ability of CRC, and si ACAD8 also inhibited the effect of CuCl_2_ (**Figure 9F**). In addition, the migration assay also supported the inhibition of CuCl_2_ and ACAD8 on the migration ability of CRC (**Figure 9G**). Oxaliplatin and 5-fluorouracil are classical chemotherapeutic agents for CRC. Our findings demonstrated that CuCl_2_ enhances the sensitivity of CRC cells to oxaliplatin and 5-fluorouracil, whereas siACAD8 attenuated the chemosensitizing effect induced by CuCl_2_ (**Supplementary Figure S6**). In conclusion, in CRC, as CRGs, ACAD8 inhibits tumor metastasis by inducing cuproptosis and suppressing cell proliferation, stemness, and migration ability.

## 4 Discussion

The metastasis of CRC is a critical factor influencing patient prognosis. Comprehensive treatments, including surgery, immunotherapy, and chemotherapy, have improved the prognosis of mCRC, but tumor heterogeneity and drug resistance still limit the effectiveness of these therapies (23). Cuproptosis, a novel copper-dependent cell death mechanism, has attracted widespread attention, but its role in metastasis of CRC remains underexplored (24). By modulating the pathways associated with cuproptosis, it may be possible to inhibit the metastasis of CRC. Copper, as a target in drug delivery systems, holds promise as a new direction for future cancer therapy (25). Given that both metastatic mechanisms and CRGs are hot research topics, exploring these areas is significant for CRC.

The combination of bioinformatics and mechanistic experiments has become an indispensable component of biological research (13, 14, 26), particularly in recent years as a popular research format. Public databases offer an open and shared platform for cancer therapy research, not only accelerating data collection and analysis but also advancing the development of precision medicine (27). By deeply mining and integrating these datasets, researchers can more efficiently identify molecular mechanisms of cancer, optimize treatment strategies, and provide robust support for personalized cancer therapy in the future (28). This study, integrating both public databases and patient sample data, proposes for the first time that ACAD8, as CRG, plays a suppressive role in the distant metastasis of CRC.

We found that ACAD8 is the only gene that satisfies the criteria for involvement in both distant metastasis and cuproptosis of CRC. Previous studies have shown that regulating ACAD8 in adipocytes can induce defects in the metabolism, ultimately leading to impaired adipocyte metabolism (29). Additionally, as a gene related to lipid metabolism, ACAD8 is influenced by the inflammatory microenvironment and can manipulate specific lipid metabolism to promote skin homeostasis (30). However, there are no reports of ACAD8 in CRC, highlighting its research potential. The low expression of ACAD8 in CRC is closely associated with, advanced TNM staging, and nerve invasion, suggesting that it may act as a suppressor of metastasis in CRC. This result was confirmed by ROC analysis, prognostic analysis, and functional experiments, though the underlying mechanisms still require further exploration. Furthermore, given the advantages of ACAD8 in diagnosing CRC and predicting prognosis, we need to focus on the combined use of ACAD8 and other biomarkers (e.g., CEA) for diagnosis of CRC (31). This validation should include both early and advanced-stage CRC patients to assess the gene’s sensitivity and specificity across different disease stages.

Immune infiltration in CRC is closely related to patient prognosis. Certain immune cell types are associated with better prognosis due to their anti-tumor effects (32). In contrast, immunosuppressive cells are often associated with drug resistance, and poor prognosis (33), which aligns with our findings that ACAD8 has a negative correlation with these immune cells. Immunotherapy for CRC is continuously evolving, and the level and type of immune cell infiltration significantly impact the effectiveness of such therapies. Our analysis reveals that ACAD8 plays an important role in immune infiltration within CRC, suggesting that ACAD8 may suppress tumor metastasis by activating immune cell infiltration. Immune cell infiltration is increasingly recognized for its role in development, progression, prognosis evaluation, and immunotherapy of CRC (34). However, it remains unclear whether ACAD8 directly influences immune cell infiltration or if other mediating factors are involved, or if immune cell infiltration affects ACAD8 expression. This is a complex issue that requires further investigation. Future research will further explore the relationship between ACAD8 and immune cell types and distribution, as well as its interactions with tumor cells, to enable more personalized and precise immunotherapy strategies.

ICIs have become a crucial component in the treatment of mCRC (35). By evaluating immune cell infiltration status, and tumor immune evasion mechanisms, researchers can develop new biomarkers to identify patients who may benefit from immunotherapy. Surprisingly, the positive correlation between immune checkpoint genes and the tumor suppressor ACAD8 was found. Tumor suppressor genes help in the immune recognition of tumor cells, thereby enhancing immune surveillance (36). The positive correlation between ACAD8 and immune checkpoint gene expression could be a result of adaptive changes in the TME (37). In some cases, tumor suppressor genes may support tumor growth by inducing immune tolerance mechanisms in tumor cells. These mechanisms may include the accumulation of immunosuppressive cells, such as Tregs and MDSCs, as well as the upregulation of immune checkpoint molecules (38). The positive correlation between ACAD8 and immune checkpoint genes may also stem from complex signaling pathways within the TME (39). Overall, the positive correlation between tumor suppressor genes and immune checkpoint genes may indicate a complex adaptive regulation of the TME, which could influence immune evasion and the tumor immune response. However, this requires further mechanistic exploration to address this complex phenomenon.

Chemotherapy resistance remains a major challenge in CRC (40). Chemotherapies, such as 5-fluorouracil, cisplatin, and irinotecan, remain essential in CRC, but the onset of resistance leads to a significant reduction in treatment efficacy and affects patient survival (41). Our study reveals that copper ions can inhibit chemotherapy resistance of CRC, and that ACAD8 enhances the sensitivity to chemotherapy drugs in CRC patients, highlighting its significant clinical translational potential as a CRGs. Previous research has shown that copper ions alter the expression of various transport proteins and enzymes, modifying the response mechanisms of cell and affecting drug tolerance (19). Additionally, cuproptosis in tumor cells may enhance their sensitivity to chemotherapy drugs by regulating energy metabolism and oxidative stress responses (42). Studying the mechanisms of cuproptosis, ACAD8, and its role in chemotherapy resistance could help overcome chemotherapy resistance in tumors and improve therapeutic outcomes.

Pathway enrichment analysis aims to identify and analyze biological pathways associated with ACAD8, by comparing experimental data with known biological pathway databases, revealing how genes act together through complex signaling and metabolic pathways in cells or organisms (43). Our pathway enrichment analysis strongly indicates that ACAD8 is a CRG. These findings suggest that ACAD8 may be involved in inhibiting invasion and metastasis of CRC through various signaling pathways, particularly those related to copper ions. The most significantly enriched pathway, “response to metal ion,” has been shown to induce cell death through different mechanisms, such as triggering biocatalysis, activating immune pathways, interfering with osmotic pressure, and generating pro-oxidant effects (44). Future research should further explore how ACAD8 regulates these signaling pathways. Further protein interaction studies suggest that ACAD8 may interact with DBT and ACADSB, among others. Previous research has confirmed that DBT is a significant prognostic marker related to the Hippo pathway, and its downregulation is activated by METTL3-mediated m6A modification (45). ACADSB, a metabolic gene, regulates total cholesterol metabolism through NAT10-dependent RNA acetylation (46). Future research will need to combine immunoprecipitation and mass spectrometry to confirm the interaction sites of ACAD8 with these proteins. Furthermore, phenotypic experiments are a core part of validating bioinformatics (47). Experimental validation demonstrated that ACAD8 can induce cuproptosis and inhibit the migration of CRC, suggesting that it can suppress distant metastatic potential of CRC. These findings strongly indicate that ACAD8 plays a crucial role in cuproptosis, with its inhibitory effects mediated through this mechanism.

Although we provide insight into the potential role and mechanisms of ACAD8 in metastasis of CRC, there are still certain limitations. First, the functional experiments mainly relied on *in vitro* cell lines and patient samples, lacking larger-scale clinical validation. Second, while we enhanced ACAD8 expression through copper ion, whether ACAD8 can effectively induce cuproptosis *in vivo* and improve CRC prognosis still requires further validation in animal models. Additionally, the role of ACAD8 in other cancers and its potential therapeutic applications also require in-depth investigation. Future studies could explore the potential therapeutic applications of ACAD8 in various cancers, particularly in the context of amino acid metabolism and its impact on tumor progression and immune response.

## 5 Conclusion

In-depth research on cuproptosis holds promise for providing more precise and effective treatment options for mCRC. This study confirms that ACAD8, as a CRG, is a novel tumor suppressor in CRC, inhibiting metastasis by inducing cuproptosis. The potential of ACAD8 in diagnosis, prognosis, enhancing immune infiltration, and increasing chemotherapy sensitivity warrants further exploration in CRC. These findings lay the foundation for individualized therapeutic strategies targeting ACAD8 to combat CRC progression and metastasis.

## Data Availability

The raw data supporting the conclusions of this article will be made available by the authors, without undue reservation.
