# Supplementary material for: Cuproptosis-related gene ACAD8 inhibits the metastatic ability of colorectal cancer by inducing cuproptosis
Source: Front Immunol. 2025 Apr 3;16:1560322. doi: 10.3389/fimmu.2025.1560322 (PMC12003318; doi:10.3389/fimmu.2025.1560322)
Supplement: Supplementary file 1 [file Table1.docx]

**Figure S1. RNA levels of ACAD8 in cancer**


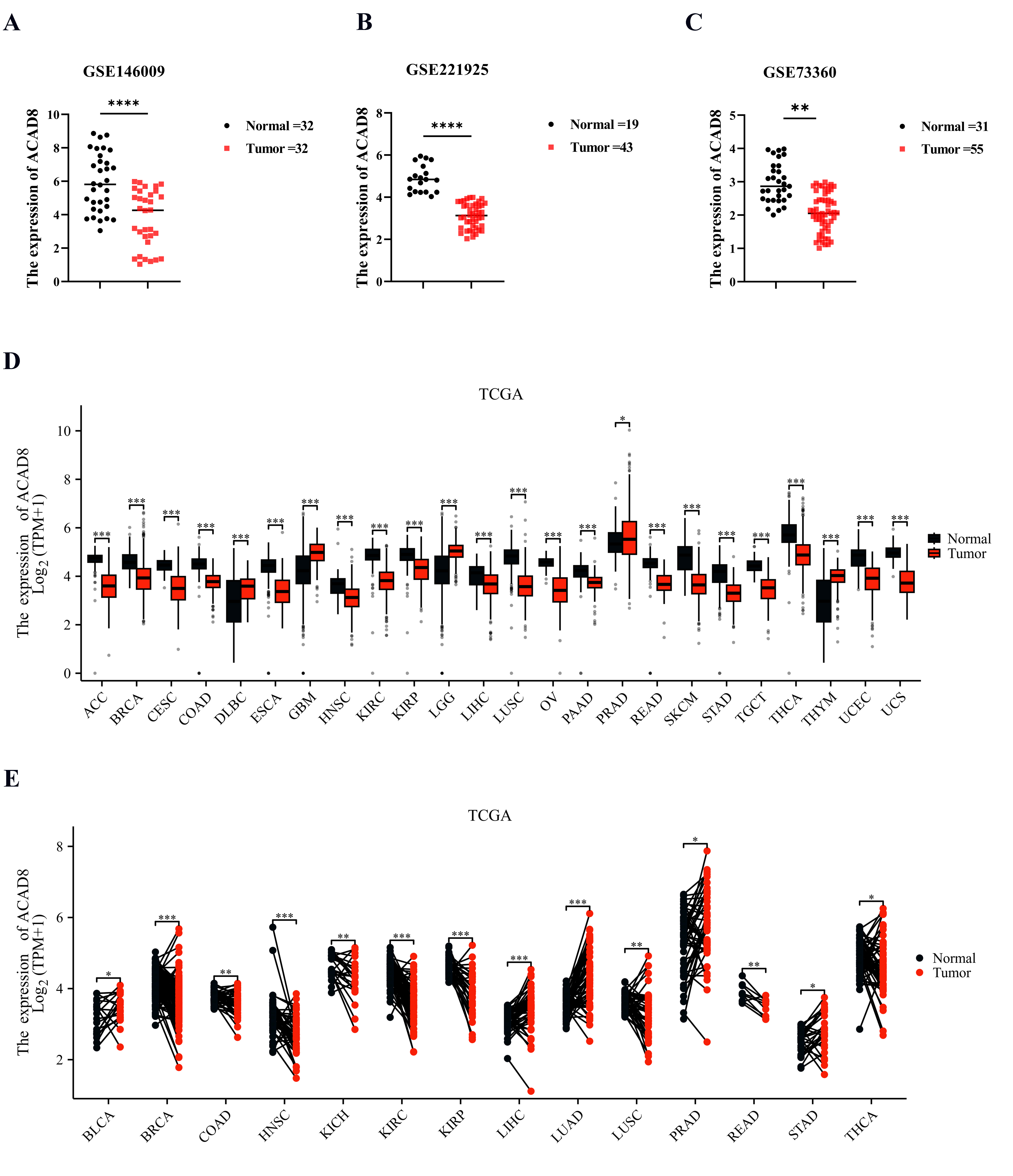


Analyzing the RNA levels of ACAD8 in CRC tissues and normal intestinal epithelial tissues based on GSE146009 (A), GSE221925 (B), and GSE73360 (C). (D) Based on TCGA, the RNA levels of ACAD8 in 24 cancer tissues and normal tissues were detected. (E) Based on TCGA, the RNA levels of ACAD8 in 14 cancer tissues and matched normal tissues were detected. (P＞0.05, ns. nonsignificant; P < 0.05 *; P < 0.01 **; P < 0.001 ***; P < 0.0001 ****; analyses were performed using Student’s t test or Wilcoxon rank-sum test, respectively).

**Figure S2. Low expression of ACAD8 indicates poor prognosis in CRC patients**


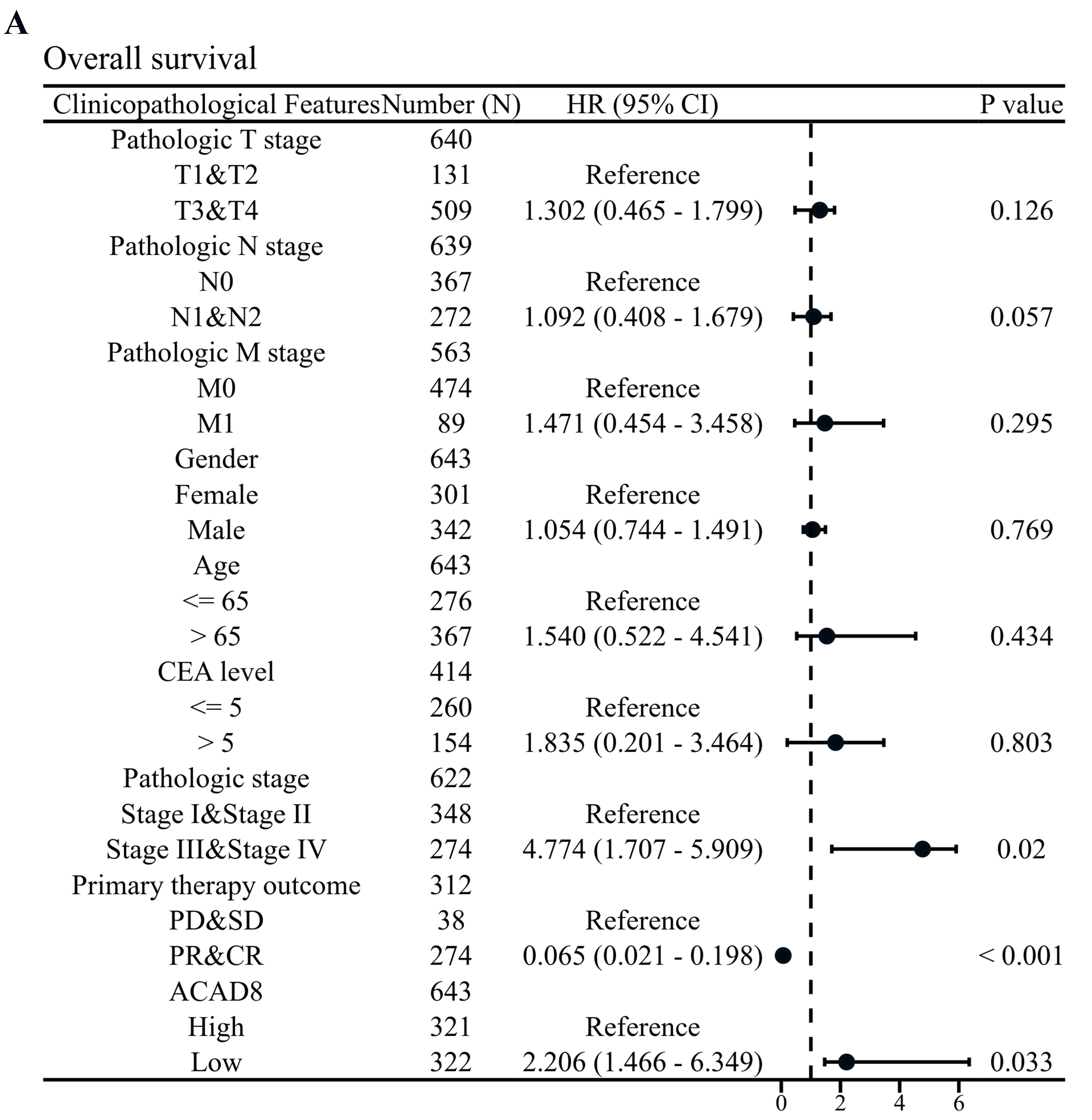


(A) A forest plot showing cox regression analysis of the impact of ACAD8 expression on OS in CRC.

**Figure S3. The relationship between expression levels of ACAD8 and immune cell infiltration**


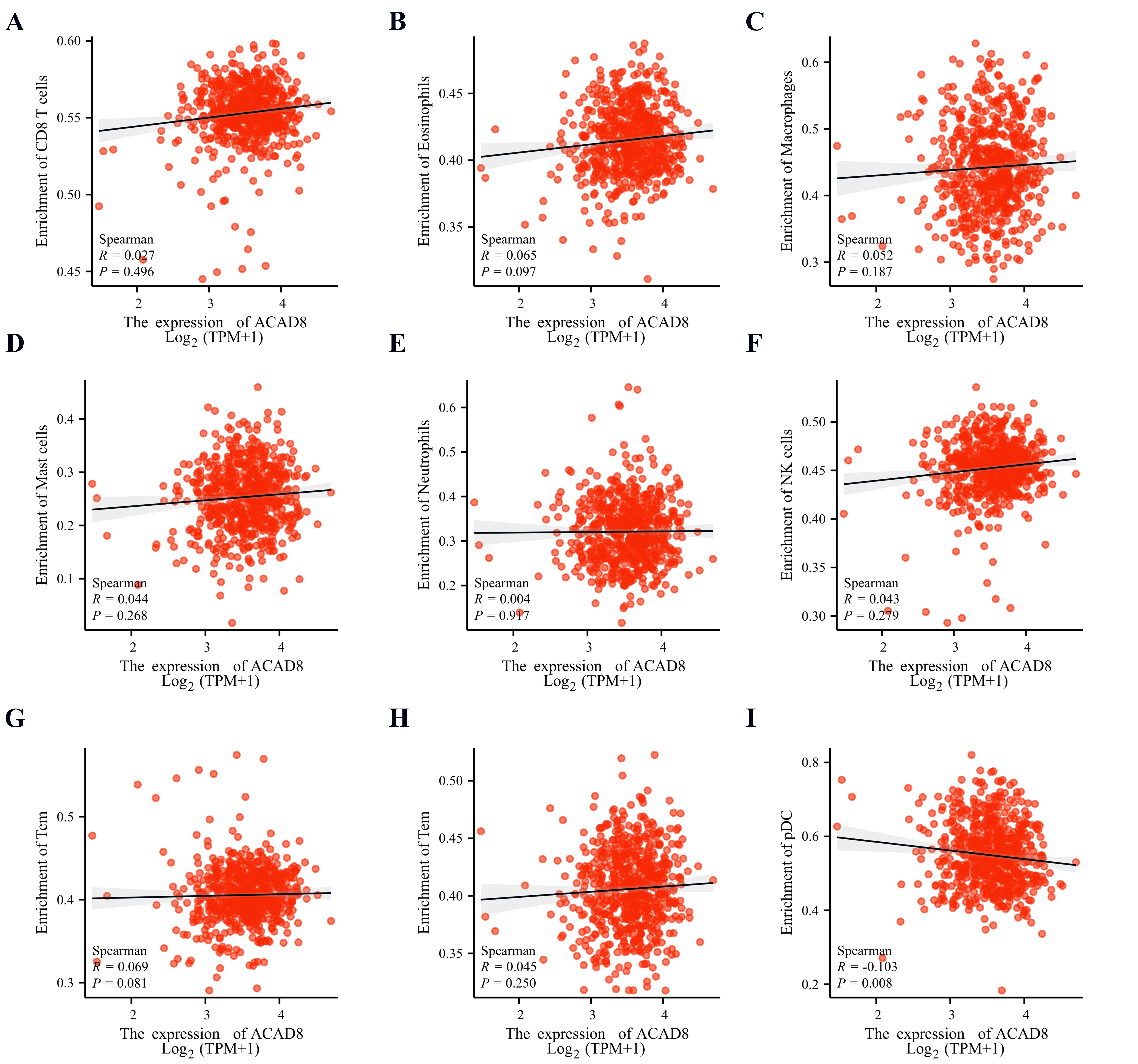


The correlation of expression levels of ACAD8 with CD8 T cell (A), Eosinophils (B), Macrophage (C), Mast cells (D), Neutrophils (E), NK cell (F), Tcm (G), Tem (H), and pDC (I).

**Figure S4. The relationship between expression levels of ACAD8 and immune cell infiltration in 33 cancer types.**


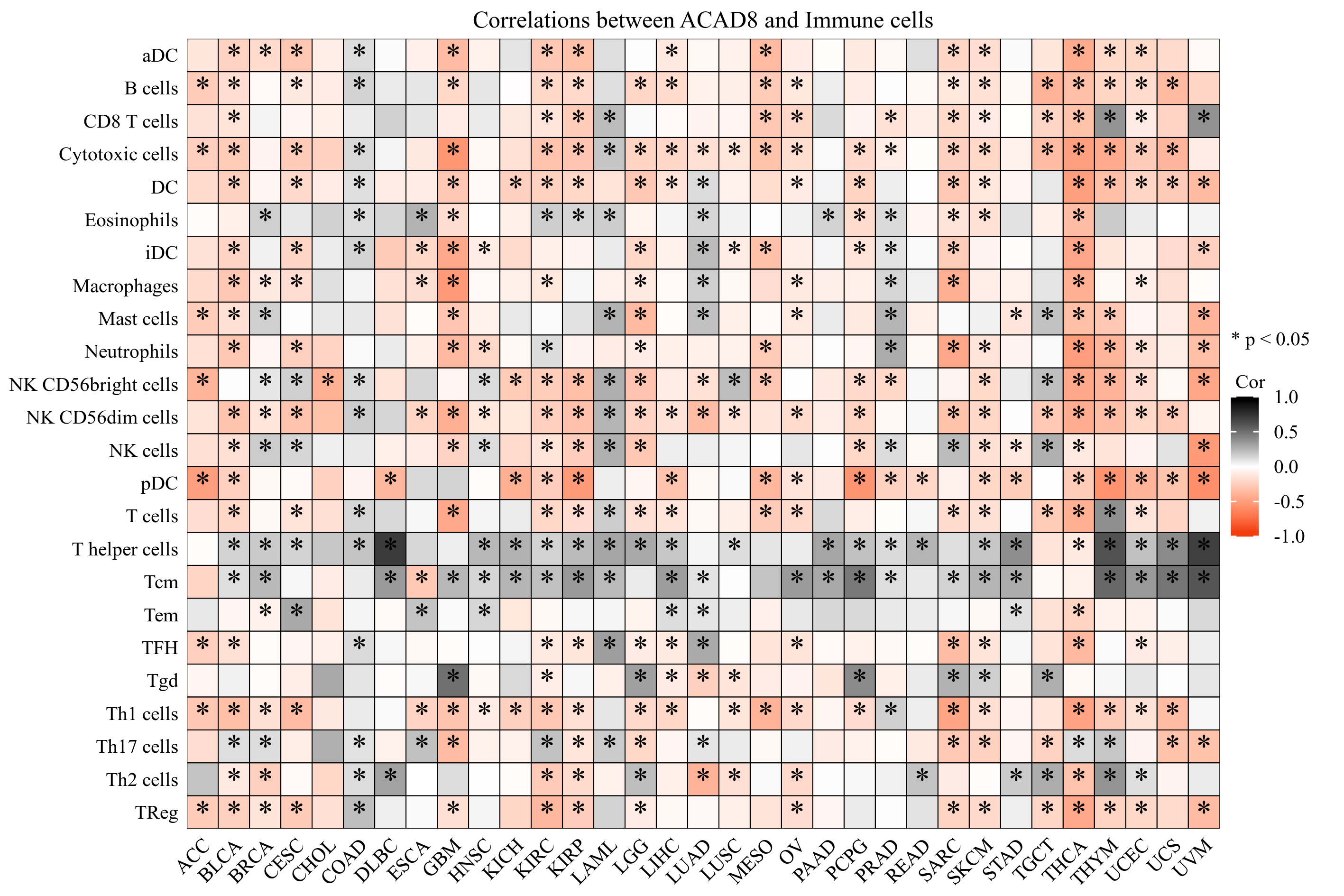


(P＞0.05, ns. nonsignificant; P < 0.05 *; P < 0.01 **; P < 0.001 ***; P < 0.0001 ****; analyses were performed using Spearman's correlation analysis).

**Figure S5. The relationship between expression levels of ACAD8 and immune checkpoint genes**


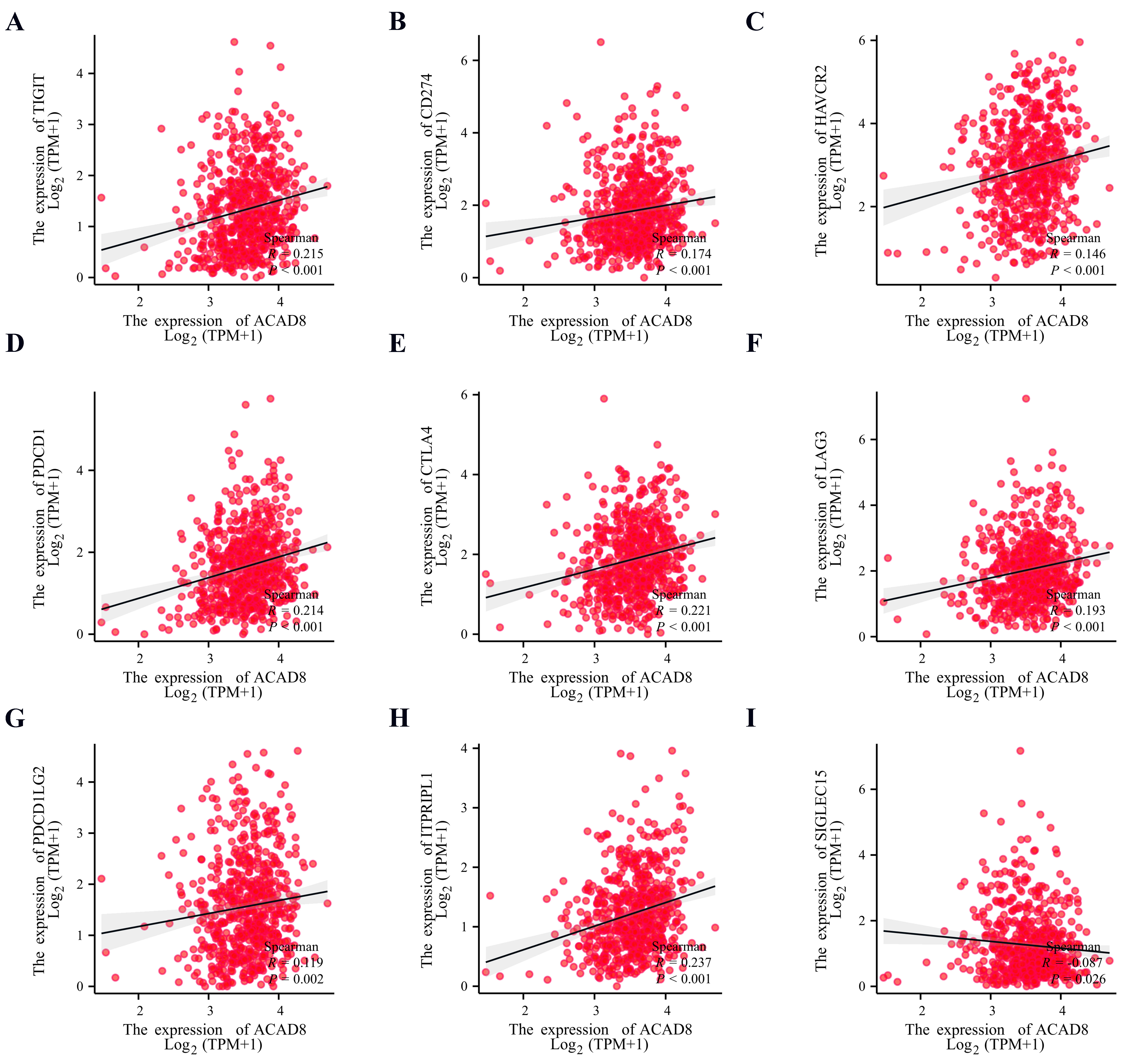


The correlation of expression levels of ACAD8 with TIGIT (A), CD274 (B), HAVCR2 (C), PDCD1 (D), CTLA4 (E), LAG3 (F), PDCD1LG2 (G), ITPRIPL1 (H), and SIGLEC15 (I).

**Figure S6. Knockdown of ACAD8 has been shown to impair chemosensitivity in CRC**


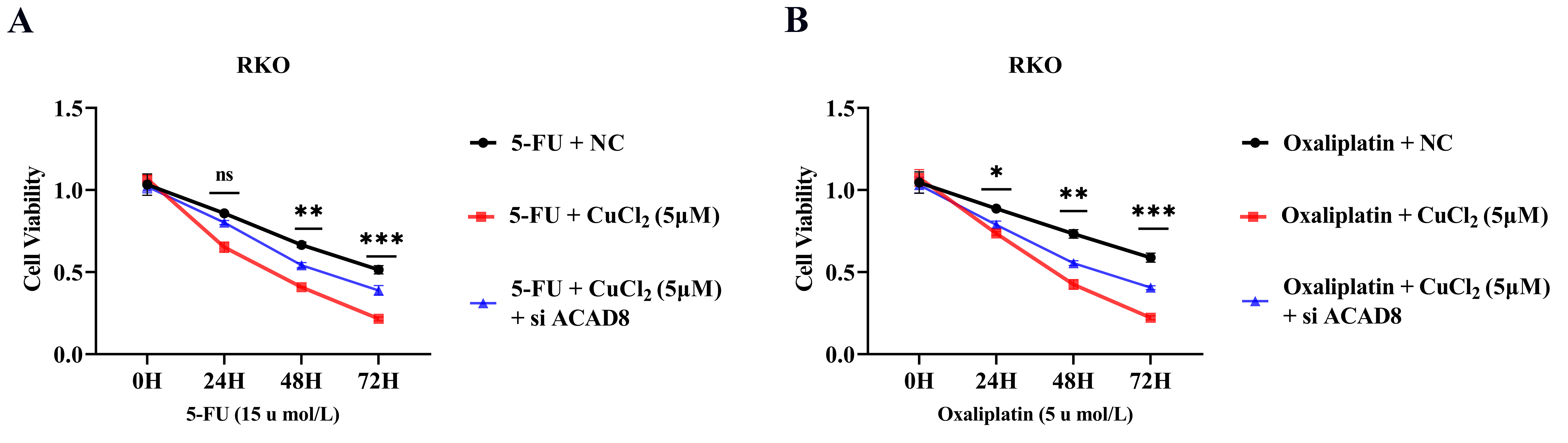


**(A–B) Chemosensitivity test based on 5-FU and Oxaliplatin.**

**Table S1 Clinic-pathological characteristics of CRC patients**

| Variables | Low ACAD8 | High ACAD8 | P^†^ |
| --- | --- | --- | --- |
| N | 40 | 40 |  |
| Age (years) |  |  |  |
| ≤65/>65 | 20/20 | 21/19 | 0.823 |
| Gender |  |  |  |
| Female/Male | 13/27 | 15/25 | 0.639 |
| ECOG PS |  |  |  |
| 0-1/2 | 20/20 | 21/19 | 0.823 |
| TNM |  |  |  |
| I-II/III | 11/29 | 17/23 | 0.160 |
| CEA at diagnosis, ng/mL |  |  |  |
| ≤5/>5 | 23/17 | 20/20 | 0.501 |

†: Pearson’s *x*^2^ test was used to analyze the basic characteristics.

ECOG PS: Eastern Cooperative Oncology Group performance status; CEA: carcinoembryonic antigen.
